# Application of TSPO-Specific Positron Emission Tomography Radiotracer as an Early Indicator of Acute Liver Failure Induced by Propacetamol, a Prodrug of Paracetamol

**DOI:** 10.3390/ijms25115942

**Published:** 2024-05-29

**Authors:** Daehee Kim, Hye Won Lee, Sun Mi Park, Ji Eun Lee, Sang Ju Lee, Bom Sahn Kim, Seung Jun Oh, Byung Seok Moon, Hai-Jeon Yoon

**Affiliations:** 1 Department of Emergency Medicine, Incheon St. Mary’s Hospital, The Catholic University of Korea, Seoul 06591, Republic of Korea; kim_dae_hee@catholic.ac.kr (D.K.); joawony@naver.com (H.W.L.); 2 Department of Emergency Medicine, College of Medicine, The Catholic University of Korea, Seoul 06591, Republic of Korea; 3 Department of Nuclear Medicine, Ewha Womans University Seoul Hospital, Ewha Womans University College of Medicine, Seoul 07804, Republic of Korea; psm9728@ewha.ac.kr (S.M.P.); kbomsahn@ewha.ac.kr (B.S.K.); 4 Department of Nuclear Medicine, Ewha Womans University Mokdong Hospital, Ewha Womans University College of Medicine, Seoul 07985, Republic of Korea; qkraltnr60n@ewha.ac.kr; 5 Department of Nuclear Medicine, Asan Medical Center, University of Ulsan College of Medicine, Seoul 05505, Republic of Korea; atlas425@amc.seoul.kr (S.J.L.); sjoh@amc.seoul.kr (S.J.O.)

**Keywords:** propacetamol, hepatic failure, [^18^F]GE180, TSPO, positron emission tomography

## Abstract

Acetaminophen overdose is a leading cause of acute liver failure (ALF), and effective treatment depends on early prediction of disease progression. ALF diagnosis currently requires blood collection 24–72 h after APAP ingestion, necessitating repeated tests and hospitalization. Here, we assessed earlier ALF diagnosis using positron emission tomography (PET) imaging of translocator proteins (TSPOs), which are involved in molecular transport, oxidative stress, apoptosis, and energy metabolism, with the radiotracer [^18^F]GE180. We intraperitoneally administered propacetamol hydrochloride to male C57BL/6 mice to induce ALF. We performed in vivo PET/CT imaging 3 h later using the TSPO-specific radiotracer [^18^F]GE180 and quantitatively analyzed the PET images by determining the averaged standardized uptake value (SUV*_av_*) in the liver parenchyma. We assessed liver TSPO expression levels via real-time polymerase chain reaction, Western blotting, and immunohistochemistry. [^18^F]GE180 PET imaging 3 h after propacetamol administration (1500 mg/kg) significantly increased liver SUV*_av_* compared to controls (*p* = 0.001). Analyses showed a 10-fold and 4-fold increase in TSPO gene and protein expression, respectively, in the liver, 3 h after propacetamol induction compared to controls. [^18^F]GE180 PET visualized and quantified propacetamol-induced ALF through TSPO overexpression. These findings highlight TSPO PET’s potential as a non-invasive imaging biomarker for early-stage ALF.

## 1. Introduction

Drug-induced liver injury (DILI) resulting from acetaminophen overdose is a significant global health concern. Acetaminophen, a widely used analgesic and antipyretic medication, is a leading cause of acute liver failure (ALF) worldwide [1,2,3]. The pathogenesis of acetaminophen-induced liver injury involves the formation of a toxic metabolite, *N*-acetyl-*p*-benzoquinone imine (NAPQI), which depletes cellular glutathione and binds to mitochondrial proteins, leading to oxidative stress, mitochondrial dysfunction, and eventually hepatocellular necrosis [4,5]. Early and accurate detection of liver injury is crucial to preventing ALF and fatal outcomes. Timely therapeutic intervention at the early stages of liver injury is vital to inhibiting further deterioration and improving patient prognosis. Therefore, there is an urgent need for sensitive and specific biomarkers to reliably identify acetaminophen-induced liver injury at its initial stages, enabling prompt medical management and reducing the burden of ALF.

However, there is currently no biomarker capable of early prediction of ALF progression in patients in the emergency room after acetaminophen overdose. Evaluation of acetaminophen-induced acute liver failure presently depends on blood biomarkers, which increase 24–72 h after ingestion, necessitating repeated testing and hospital admission [6,7]. All patients suspected of consuming excess acetaminophen or potentially overdosing must be admitted for observation, which is a considerable waste of healthcare resources. Furthermore, the inability to predict early progression to ALF makes it difficult to prepare for a liver transplant, if needed. There is a need to develop diagnostic techniques that can enable early prediction of liver failure progression in patients with acetaminophen-induced liver injury.

The translocator protein (TSPO), formerly known as the peripheral benzodiazepine receptor (PBR), is a mitochondrial membrane protein that has gained significant attention in the neurology, oncology, and immunology fields [8,9]. TSPO expression can be upregulated in response to cellular stress, including oxidative stress and inflammation, potentially representing an adaptive response to cellular injury aimed at regulating mitochondrial function and cellular survival [10,11,12]. However, sustained TSPO overexpression may contribute to chronic inflammation and tissue damage in inflammatory diseases. TSPO overexpression in inflammatory diseases has led to its consideration as a potential diagnostic and therapeutic target, with TSPO ligands, such as radiotracers used in positron emission tomography (PET) imaging, enabling non-invasive, in vivo inflammation assessment.

Considering the oxidative stress caused by NAPQI, ROS production, and glutathione depletion in the early pathophysiology of acetaminophen-induced liver injury, which leads to hepatocellular necrosis, TSPO-targeted PET imaging shows promise for early evaluation of drug-induced liver injury. Various TSPO-specific and selective radiotracers have been developed, including (4S)-*N*,*N*-diethyl-9-[2-[^18^F]fluoroethyl]-5-methoxy-2,3,4,9-tetrahydro-1*H*-carbazole-4-carboxamide ([^18^F]GE180, flutriciclamide). This is a third-generation radioligand that recognizes TSPO with high affinity and specific binding [13,14,15]. We previously investigated the feasibility of using the TSPO-specific radiotracer [^18^F]GE180 to visualize acetaminophen-induced liver injury using PET. We observed increased liver enzyme levels accompanied by elevated hepatic uptake on TSPO-targeted GE180 PET imaging 24 h after acetaminophen overdose in rats. In the present study, we aimed to evaluate the applicability of GE180 PET for early ALF diagnosis and prognosis prediction by administering a lethal dose of propacetamol (a prodrug of acetaminophen).

## 2. Results

### 2.1. Dose-Dependent Mouse Mortality Induced by Propacetamol

**Figure 1:**
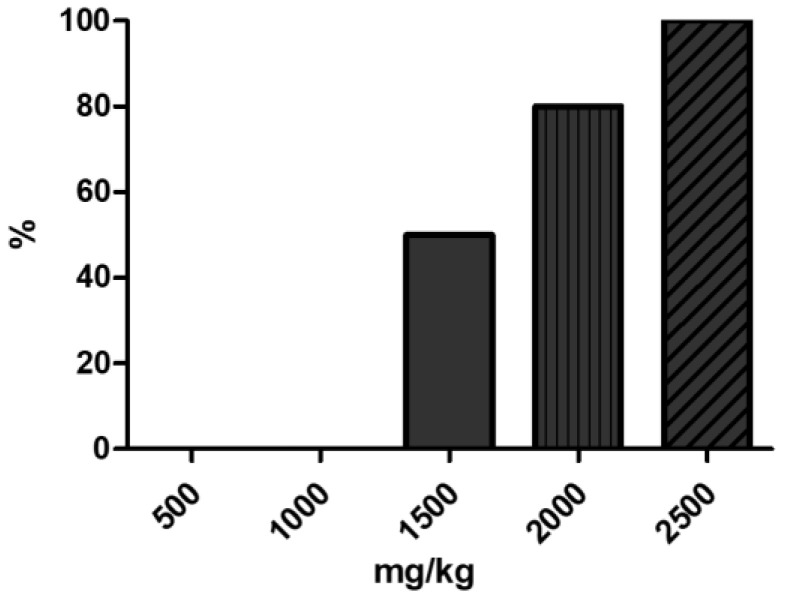
Changes in mouse mortality with increasing propacetamol doses; n = 3 for 500 mg/kg, n = 3 for 1000 mg/kg, n = 4 for 1500 mg/kg, n = 5 for 2000 mg/kg, and n = 2 for 2500 mg/kg.

To determine the appropriate lethal dose of propacetamol, we administered doses ranging from 500 mg/kg to 2500 mg/kg and observed the mice for 24 h. No deaths were observed at doses up to 1000 mg/kg. Among the four mice injected with 1500 mg/kg, two died (50% mortality rate), while four out of five mice injected with 2000 mg/kg died (80% mortality rate). Finally, all mice injected with 2500 mg/kg died, showing a 100% mortality rate (Figure 1).

### 2.2. Dose- and Time-Dependent Alterations in Serum Biochemistry Induced by Propacetamol

**Figure 2:**
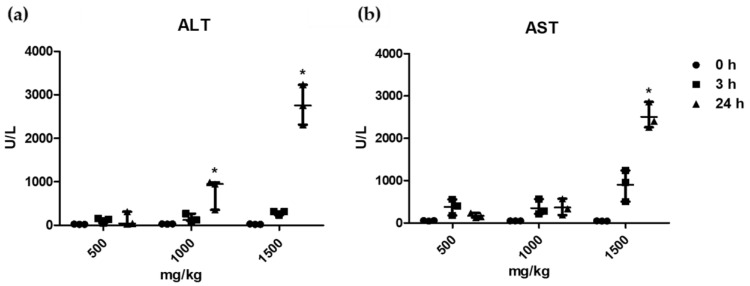
Changes in serum biomarker levels for hepatocellular injury. (**a**) ALT levels for each dose group before, 3 h after, and 24 h after propacetamol administration. (**b**) AST levels for each dose group before, 3 h after, and 24 h after propacetamol administration; n = 3 for 500 mg/kg, n = 3 for 1000 mg/kg, and n = 3 for 1500 mg/kg. Data are expressed as median values with interquartile ranges; * *p* < 0.05 by the Friedman test, which is a non-parametric statistical test used for repeated measures within fixed doses of propacetamol.

To assess the levels of ALT and AST in response to propacetamol dosage and time, we administered doses ranging from 500 mg/kg to 1500 mg/kg, which exhibited a 50% mortality rate. We continuously conducted blood sampling at 3 h and 24 h post-administration. ALT levels did not significantly change over time following the 500 mg/kg injection. At doses of 1000 mg/kg and 1500 mg/kg, no significant alterations were observed up to 3 h, but a significant increase was detected at 24 h (Figure 2a). AST levels showed no significant change over time up to 1000 mg/kg. However, upon administration of 1500 mg/kg, a significant increase in AST levels was noted at 24 h (Figure 2b).

### 2.3. Time-Dependent Hepatocyte Necrosis Induced by a Lethal Dose of Propacetamol

**Figure 3:**
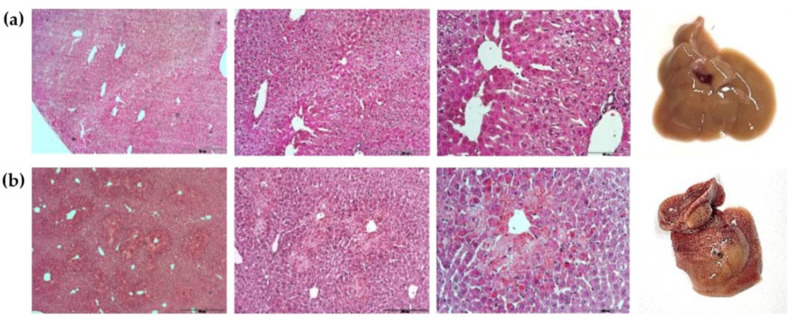
Microscopic and macroscopic liver tissue changes. (**a**) Liver tissue 3 h post-administration of 1500 mg/kg propacetamol. The first three panels (from left to right) show histological sections of liver tissue stained with hematoxylin and eosin (H&E) at different magnifications (scale bar: 50 μm for 40×, 200 μm for 100×, and 100 μm for 200×). These sections show normal liver tissue architecture with no apparent signs of hepatocyte necrosis. The fourth panel is a macroscopic liver examination 3 h post-administration, which also appears normal. (**b**) Liver tissue 24 h post-administration of 1500 mg/kg propacetamol. The first three panels depict histological sections at different magnifications (scale bar: 50 μm for 40×, 200 μm for 100×, and 100 μm for 200×), showing widespread centrilobular necrosis (pink areas). The fourth panel exhibits extensive petechial hemorrhages as small pinpoint bleeding spots, indicating severe liver injury.

To determine hepatic necrosis development over time following 1500 mg of propacetamol, we euthanized mice 3 and 24 h post-administration and assessed gross and hematoxylin and eosin (H&E) histological changes (n = 3, each time point). At 3 h post-injection of 1500 mg/kg propacetamol, both macroscopic examination and microscopic H&E staining revealed normal liver tissue. However, at 24 h, macroscopic examination exhibited extensive petechial liver hemorrhage, while H&E staining revealed widespread necrosis (Figure 3).

### 2.4. [^18^F]GE180 PET Findings in Propacetamol-Induced Acute Liver Failure Model

**Figure 4:**
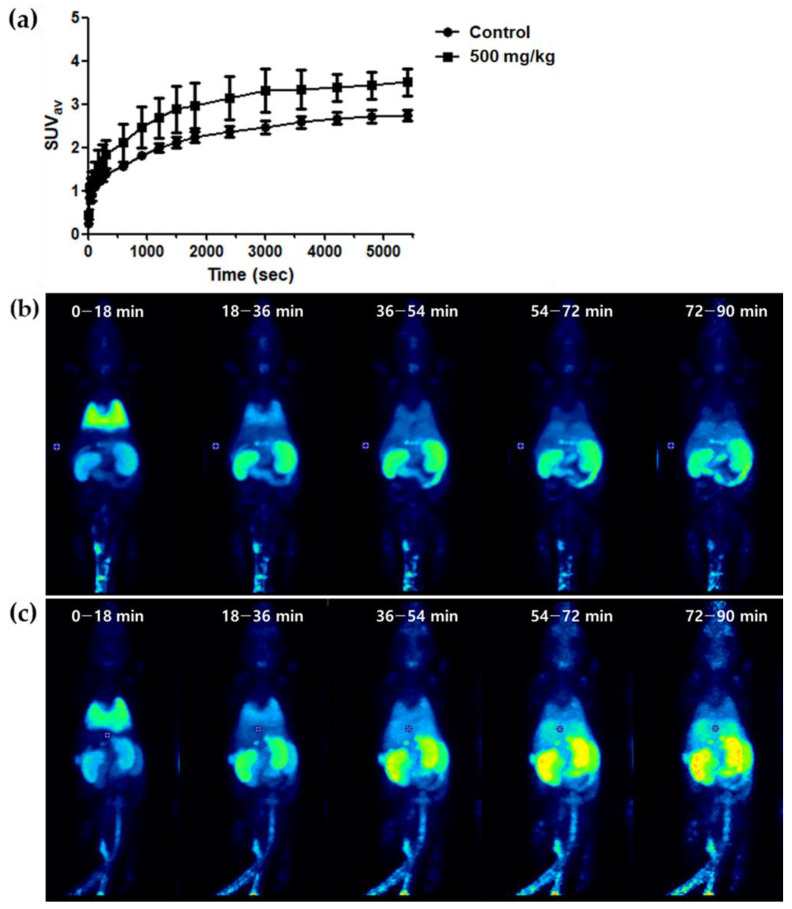
PET results in list mode. (**a**) Time–activity curve for liver SUV*_av_* during a 90 min list-mode PET acquisition with [^18^F]GE180. The PET data were acquired immediately after a bolus injection of [^18^F]GE180 and reconstructed into 19 frames (4 × 15 s, 4 × 60 s, 5 × 300 s, 6 × 600 s). To generate the time–activity curve, the same volume of interest (VOI) was positioned on the liver parenchyma for all frames. The curve demonstrates that the liver SUV*_av_* plateaued around 60 min post-injection and remained stable. The time–activity curve analysis confirmed that the SUV*_av_* values plateaued and were maintained after 60 min, suggesting that the [^18^F]GE180 uptake in the liver reached a steady state approximately 1 h post-injection. The 500 mg/kg dose shows a slightly higher SUV*_av_* throughout the 90 min acquisition period than the control. However, both curves plateaued around 60 min post-injection, indicating that propacetamol administration did not significantly alter [^18^F]GE180 liver uptake kinetics compared to the control. (**b**,**c**) Time–activity curves from a 90 min list-mode acquisition reconstructed into 5 frames following injection of [^18^F]GE180 in the control (**b**) and 500 mg/kg propacetamol-treated (**c**) mice. In both groups, the initial frame (0–18 min) shows predominant tracer uptake in the lungs, which rapidly declines in the subsequent frames. However, in the propacetamol-treated group, hepatic uptake progressively increases, becoming more prominent compared to the control group from the 4th frame (54–72 min) onwards and persisting in the final frame (72–90 min).

Our study objective was to assess the effectiveness of [^18^F]GE180 PET in detecting early-stage hepatic failure induced by propacetamol. First, PET imaging was performed 3 h after administering 500 mg/kg of propacetamol. The conditions were relatively mild to ensure the subject could endure prolonged anesthesia and to allow for successful image acquisition during the 90 min dynamic scan. Utilizing a time–activity curve for the 90 min post-[^18^F]GE180 injection (Figure 4), we conducted a static mode acquisition for 20 min, starting at 60 min, when the plateau phase was reached.

**Figure 5:**
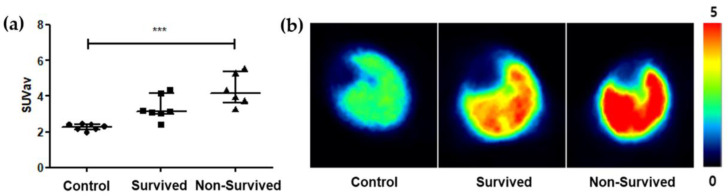
Comparison of [^18^F]GE180 PET findings. (**a**) Comparison of liver SUV*_av_* measured from [^18^F]GE180 PET between the control, survived, and non-survived groups. The survived and non-survived groups were subgroups from a study of propacetamol-induced hepatic failure, where 1500 mg/kg of propacetamol was administered to mice. [^18^F]GE180 PET imaging was performed 3 h post-administration, and mouse survival was monitored for 48 h. Mice were then classified into survived or non-survived groups based on their survival outcome. The control group had the lowest liver SUV*_av_*, while the non-survived group showed a significantly higher liver SUV*_av_* compared to the control group; n = 7 for control, n = 7 for survived, and n = 6 for non-survived. Data are expressed as median values with interquartile ranges; *** *p* < 0.001 by the Kruskal–Wallis test. (**b**) Representative axial view slices of the liver from [^18^F]GE180 PET imaging in control, survived, and non-survived mice. The color scale represents the SUVs of the PET tracer [^18^F]GE180 in the liver. In the control group (left image), the liver shows a relatively low uptake of the tracer, as indicated by the blue and green colors. In the survived group (middle image), the liver exhibits slightly higher tracer uptake compared to the control group, with some areas showing yellow and orange colors. In the non-survived group (right image), the liver displays substantially higher tracer accumulation, as evidenced by the predominantly red and orange colors, indicating increased uptake of [^18^F]GE180.

We administered 1500 mg/kg of propacetamol to 13 mice, which resulted in a 50% mortality rate, with no significant elevation in ALT levels or evidence of microscopic hepatocyte necrosis, and acquired [^18^F]GE180 PET images 3 h after propacetamol administration. Compared to the control group, the liver SUV*_av_* in the group receiving the lethal dose of propacetamol significantly increased (median of 2.3, with an IQR of 2.14–2.42 for the controls; median of 3.76, with an IQR of 3.09–4.35 for the ALF group; *p*-value = 0.001 by Mann–Whitney test; Figure S1). During the 48 h observation period, six of the thirteen mice died while seven survived. The liver SUV*_av_* in the non-survived group demonstrated a significant increase compared to the survived group (median of 3.13, with an IQR of 3.02–4.15 for the survived group; median of 4.15, with an IQR of 3.64–5.37 for the non-survived group; *p*-value = 0.045 by Mann–Whitney test; Figure S1). When comparing the three groups, the control group had the lowest liver SUV*_av_*, while the non-survived group showed a significantly higher liver SUV*_av_* compared to the control group (Figure 5).

### 2.5. mRNA and Protein Expression of TSPO in Propacetamol-Induced Hepatic Failure Model

**Figure 6:**
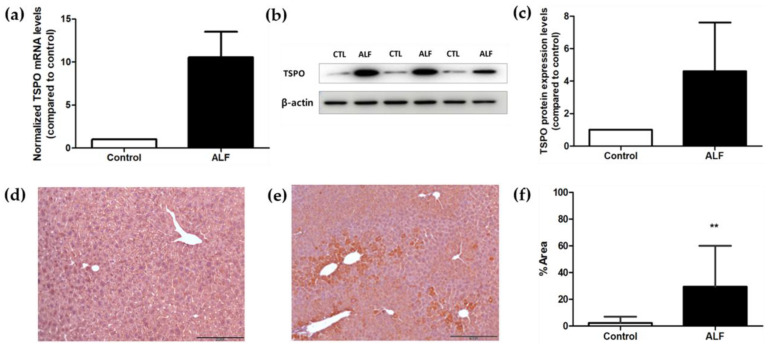
Identification of ex vivo TSPO expression at ALF in mRNA and protein levels. (**a**) The real-time PCR analysis result shows that mRNA levels of the TSPO were approximately 10-fold higher in the liver tissue of the group with acute liver failure (ALF) induced by propacetamol compared to the control group at 3 h after induction. The bar graph visually represents this substantial increase in TSPO mRNA expression in the ALF model group relative to the control. (**b**,**c**) The Western blotting analysis shows that protein levels of the TSPO were approximately 4-fold higher in the liver tissue of the ALF compared to the control group 3 h after induction. The bar graph visualizes this substantial increase in TSPO protein expression in the ALF model group relative to the control. (**d**) Representative images of TSPO immunohistochemical (IHC) staining in the liver tissues of the control group show no brown spots, indicating minimal TSPO expression in normal liver tissues (scale bar: 200 μm for 100× magnification). (**e**) Representative images of TSPO IHC staining in the liver tissues of the acute liver failure (ALF) group 3 h after induction show extensive brown spots primarily localized in the centrilobular regions, indicating higher TSPO protein expression (scale bar: 200 μm at 100× magnification). (**f**) Quantification of the immunohistochemical brown staining for TSPO in the control and ALF groups using ImageJ revealed a substantially larger stained area in the ALF group. Data are expressed as median values with interquartile ranges. ** *p* < 0.01 by Mann–Whitney test.

Real-time polymerase chain reaction (PCR) results 3 h after induction in the propacetamol-induced liver failure group showed that TSPO gene expression in the liver increased approximately 10-fold compared to the control group (Figure 6a). Western blotting results 3 h after induction in the propacetamol-induced liver failure group showed an approximately 4.0-fold increase in TSPO protein expression in the liver compared to the control group (Figure 6b,c, Figures S2 and S3). Immunohistochemistry results 3 h after the induction of propacetamol-induced liver failure showed significantly higher TSPO protein expression in the liver failure group compared to the control group (Figure 6d–f).

## 3. Discussion

Acetaminophen overdose is a leading cause of ALF, and early prediction of fatal disease progression supports prompt intervention, aids treatment planning, allows for efficient medical resource allocation, reduces complications, and ultimately, improves patient outcomes through enhanced survival rates. In this study, we aimed to assess the feasibility of earlier ALF diagnosis using PET imaging of TSPO, a key transmembrane protein involved in various cellular processes, such as molecular transport, oxidative stress, apoptosis, and energy metabolism. We induced ALF in C57BL/6 mice using a propacetamol overdose model. We performed in vivo PET imaging with the TSPO-specific radiotracer [^18^F]GE180 3 h after ALF induction. We then compared [^18^F]GE180 hepatic uptake between the control and ALF groups and among different prognosis groups. These findings were then validated through ex vivo TSPO mRNA and protein expression analyses. We combined non-invasive PET imaging with ex vivo analyses to investigate the potential of TSPO as an early biomarker and assess the feasibility of [^18^F]GE180 PET as a tool for the early diagnosis and prognostic prediction of acetaminophen-induced liver failure.

In this study, we employed propacetamol, an acetaminophen prodrug, to induce ALF caused by acetaminophen overdose. Propacetamol is more water-soluble than acetaminophen, facilitating its administration in animal studies. While many studies have reported that acetaminophen doses ranging from 200 to 400 mg/kg are sufficient to induce significant liver toxicity in mice [16,17], Liou et al. demonstrated that 1200 mg/kg resulted in 50% survival in C57BL/6 mice [18]. We administered propacetamol at doses ranging from 500 mg/kg to 2500 mg/kg and observed 50% survival at 1500 mg/kg, which we established as the lethal dose for assessing PET imaging in acetaminophen-induced ALF.

ALT, a blood biomarker of liver injury, is currently measured through repeated blood tests along with other liver enzymes to predict ALF [6]. However, this approach has limitations. Our results showed that even with propacetamol doses exceeding 1000 mg/kg, both ALT and AST levels were not significantly elevated 3 h post-administration, requiring 24 h to display abrupt increases. A 1500 mg/kg propacetamol dose, which resulted in a 50% mortality rate, induced significant ALT and AST elevations 24 h post-administration. The lack of significant liver enzyme elevations at 3 h followed by abrupt increases at 24 h is consistent with the histological findings, which showed no hepatocyte microscopic necrosis at 3 h, but extensive necrosis 24 h post-administration of a lethal dose of propacetamol. These findings highlight the need for alternative methods to detect ALF, as the current approach of monitoring liver enzymes through repeated blood tests may not offer timely prediction, particularly in the critical early stages of liver injury.

The results of the present study demonstrate the potential of [^18^F]GE180 PET imaging as an early diagnostic tool for detecting propacetamol-induced ALF. At 3 h post-administration of a lethal dose of propacetamol (1500 mg/kg), PET imaging revealed a significant increase in liver SUV*_av_* compared to the control group, despite the absence of significant elevations in ALT levels or microscopic evidence of hepatocyte necrosis. These findings suggest that [^18^F]GE180 PET imaging can detect early changes in liver function before the onset of significant liver enzyme elevations or histological evidence of necrosis, which typically occurs at later stages of liver injury. Furthermore, mice that did not survive the 48 h observation period exhibited a significantly higher liver SUV*_av_* than those that survived, suggesting that [^18^F]GE180 PET imaging may have prognostic value in ALF.

Recent studies have explored the application of TSPO PET imaging in DILI, highlighting its potential for the detection and monitoring of liver damage. Our findings are consistent with previous reports that have demonstrated the utility of TSPO PET imaging in various models of liver injury. Hatori et al. showed the feasibility of using TSPO PET imaging with [^18^F]FEDAC to non-invasively visualize acute liver damage in a rat model induced by cycloheximide [19]. They found that [^18^F]FEDAC uptake increased in the damaged livers compared to the controls, and this uptake correlated with the severity of liver injury. Another study by Hatori et al. demonstrated the potential of [^18^F]FEDAC PET imaging to non-invasively monitor liver fibrosis progression in a rat model of carbon tetrachloride (CCl4)-induced liver injury [20]. They found that PET imaging with [^18^F]FEDAC could track the progression of liver damage, with increased radiotracer uptake correlating with liver injury severity and duration. In our previous study, we evaluated the feasibility of using [^18^F]GE180 PET imaging to visualize and quantify paracetamol-induced liver injury in a rat model [21]. We found a significant increase in [^18^F]GE180 hepatic uptake in the injury group compared to the control group, which correlated well with ex vivo TSPO expression and liver injury serum markers. This study, along with the findings of Hatori et al., underscores the versatility of TSPO PET imaging in assessing various types of liver injury and supports its potential clinical application in the non-invasive evaluation of liver diseases.

In our previous study [21], we induced liver injury in Sprague Dawley rats by intraperitoneally administering propacetamol at a dose of 3 g/kg body weight, based on a previous study in a rat model [22]. However, we used a sub-lethal dose of propacetamol, which did not result in mortality due to ALF. In contrast, the current study employed a lethal dose of propacetamol (1500 mg/kg), which resulted in a 50% mortality rate. Another key difference between the two studies is the timing of the PET imaging. In our previous study, [^18^F]GE180 PET imaging was performed 24 h after propacetamol administration, which allowed for an assessment of liver injury severity through the correlation between [^18^F]GE180 hepatic uptake and the observed liver enzyme elevations. In comparison, the current study focused on the potential of TSPO PET imaging in detecting liver damage at an early stage by examining the differences in hepatic uptake between the ALF and control groups 3 h post-injury. This study investigated the prognostic value of TSPO PET imaging in predicting ALF outcomes by comparing the hepatic uptake between the survived and non-survived groups 3 h post-injury, before any significant elevations in liver enzymes or histological evidence of necrosis. The increased [^18^F]GE180 liver uptake was supported by TSPO gene upregulation and protein expression in the liver, as demonstrated by real-time PCR, Western blotting, and immunohistochemistry analyses.

In the early stages of acetaminophen overdose-induced liver injury, increased TSPO expression may be attributed to inflammatory response and cellular stress. Although the exact functions of TSPO have not been fully elucidated, it is involved in various cellular processes, including steroidogenesis, mitochondrial function, and inflammation [8,9,23]. In our previous study [21], immunohistochemistry revealed particularly high TSPO expression in the hepatocytes of those with acetaminophen-induced liver injury with controls. TSPO expressions were observed in hepatocytes of zone 3, which is the primary site of hepatocyte necrosis and a histopathological indicator of acetaminophen-induced liver injury. In the current study, TSPO IHC staining in the liver tissues of the ALF group 3 h after induction showed extensive brown spots, primarily localized in the centrilobular regions, indicating higher TSPO protein expression in this group compared to the controls. Although TSPO is constitutively expressed at low levels in hepatocytes under normal conditions [9], its expression can be upregulated in response to various stimuli, including oxidative stress and inflammation [24]. The increased TSPO expression in hepatocytes during acetaminophen-induced liver injury may be a consequence of the direct toxic effects of acetaminophen metabolites, such as NAPQI, including mitochondrial dysfunction and oxidative stress [4,10,11]. The inflammatory mediators released by activated immune cells may also contribute to upregulated TSPO expression in hepatocytes [25].

Consequently, our study demonstrates the feasibility and potential utility of [^18^F]GE180 PET imaging in the early detection and evaluation of acetaminophen overdose-induced ALF. The significant increase in [^18^F]GE180 hepatic uptake observed in the ALF group 3 h post-induction, before substantial elevations in liver enzymes or histological evidence of necrosis, highlights the sensitivity of TSPO PET imaging in detecting early-stage liver injury. Furthermore, the differences in uptake between the survived and non-survived groups suggest that TSPO PET imaging may be a promising tool for assessing the severity and predicting the outcome of acetaminophen-induced liver injuries. The increased TSPO expression observed primarily in hepatocytes during the early stages of liver injury underscores that this imaging approach captures the cellular response to drug-induced toxicity before overt tissue damage occurs. As a non-invasive means of early detection and monitoring, [^18^F]GE180 PET imaging may facilitate timely therapeutic interventions and improve clinical management in cases of acetaminophen overdose-induced liver injury.

Although our findings demonstrate the feasibility and potential usefulness of [^18^F]GE180 PET imaging in the early detection and evaluation of ALF induced by acetaminophen overdose, the clinical translatability of these findings may vary. Therefore, further validation experiments using larger numbers of animals, higher-order animal models, and ultimately, validation in human subjects, are needed.

The potential influence of TSPO polymorphisms on [^18^F]GE180 binding affinity and liver uptake is a major concern. Although [^18^F]GE180 is a third-generation TSPO ligand designed to be less sensitive to polymorphisms, some studies have reported varying binding affinities in individuals with different TSPO genotypes [13,15]. While the impact of TSPO polymorphisms on [^18^F]GE180 uptake may be masked by the substantial increase in TSPO expression during liver injury, future clinical studies should investigate the relationship between [^18^F]GE180 hepatic uptake and TSPO expression in genotyped individuals. In addition, the results obtained from this preclinical study may not directly translate to clinical settings. Further research is necessary to assess the utility of [^18^F]GE180 PET imaging in patients with acetaminophen-induced liver injury and to establish its role in the early diagnosis and prognostic assessment of ALF.

## 4. Materials and Methods

### 4.1. Synthesis of [^18^F]GE180

We prepared the TSPO-specific targeting radiotracer, [^18^F]GE180, according to previously established methods, starting from the mesylate precursor, (S)-2-(4-(diethylcarbamoyl)-5-methoxy-3,4-dihydro-1H-carbazol-9(2*H*)-yl)ethyl methanesulfonate, via Kryptofix-mediated nucleophilic aliphatic substitution with fluorine-18 [21]. The isolated product exhibited a non-decay-corrected radiochemical yield of 35.4 ± 3.8% (n = 27), calculated from trapped radioactivity on a QMA carbonate plus light cartridge. The radiochemical purity exceeded 99%, as analyzed by analytical high-performance liquid chromatography (HPLC). The molar activity was 178 ± 54 GBq/μmol, with approximately 99% radiochemical purity, as confirmed by analytical HPLC, utilizing UV-254 nm absorption at the preparation endpoint.

### 4.2. Mice and Induction of Propacetamol-Induced Acute Liver Failure (ALF)

All animals were maintained in accordance with the National Research Council guidelines for the care and use of laboratory animals (revised in 1996). We purchased all specific pathogen-free male C57BL/6 mice (weight 24.4 ± 0.8 g, 10- to 11-week-old) from Orient Bio Inc. (Seongnam, Republic of Korea). Mice were acclimated in an animal room under controlled temperature (21 °C) and a 12 h light–dark cycle. The ALF mouse model was induced as described previously [18]. Mice were fasted for 12 h, followed by intraperitoneal administration of a vehicle or propacetamol hydrochloride (Denogan^®^, Yungjin Pharm. Co., Ltd., Seoul, Republic of Korea).

### 4.3. Mouse Numbers

The numbers of mice used for analysis were the following: (1) dose-dependent mouse mortality induced by propacetamol—n = 3 for 500 mg/kg; n = 3 for 1000 mg/kg; n = 4 for 1500 mg/kg; n = 5 for 2000 mg/kg; and n = 2 for 2500 mg/kg; (2) dose- and time-dependent alterations in serum biochemistry induced by propacetamol—n = 3 for 500 mg/kg; n = 3 for 1000 mg/kg; and n = 3 for 1500 mg/kg; (3) time-dependent hepatocyte necrosis induced by propacetamol—n = 3 for 3 h post-injection and n = 3 for 24 h post-injection; (4) list-mode PET acquisition—n = 3 for control group and n = 3 for 500 mg/kg; (5) static mode PET acquisition—n = 7 for control and n = 13 for 1500 mg/kg; (6) ex vivo real-time polymerase chain reaction analysis—n = 3 for control and n = 3 for 1500 mg/kg; (7) ex vivo Western blotting analysis—n = 3 for control and n = 3 for 1500 mg/kg; and (8) immunohistochemistry analysis—n = 6 for control and n = 11 for 1500 mg/kg.

### 4.4. In Vivo [^18^F]GE180 PET Imaging

We conducted [^18^F]GE180 PET/CT using a dedicated small animal PET/CT scanner (NanoPET/CT; Mediso Medical Imaging Systems, Budapest, Hungary) with an axial field of view of 8.0 cm and a transaxial field of view of 10.0 cm. Mice were anesthetized with 1.5% isoflurane and 10.0 ± 2.8 MBq/0.2 mL of [^18^F]GE180 administered intravenously.

For list-mode PET acquisition, we began scans immediately after a bolus injection of [^18^F]GE180 over 90 min, whereas scans for static-mode PET acquisition started 60 min after the [^18^F]GE180 injection, lasting 20 min. We performed the CT scans for anatomical co-registration of PET images. Images were reconstructed using a three-dimensional ordered subset expectation maximization algorithm and CT-based attenuation correction. We also reconstructed 90 min list-mode data into 19 frames (4 × 15 s, 4 × 60 s, 5 × 300 s, and 6 × 600 s) to generate time–activity curves.

### 4.5. PET Image Analysis

For quantitative analysis, we converted all PET and CT images into the Digital Imaging and Communications in Medicine (DICOM) format and analyzed them using PMOD 4.2 software (PMOD Technologies Ltd., Zurich, Switzerland). Utilizing maximum intensity projection (MIP) images as a guide, we determined the averaged standardized uptake value (SUV*_av_*) by positioning a 3D volume of interest (VOI) on the liver parenchyma under the supervision of an experienced nuclear medicine specialist. SUV was computed in pixels as the ratio of tissue radioactivity concentration to the injected dose divided by body weight.

### 4.6. Serum Biochemistry Analysis

We collected whole blood samples from the infraorbital vein before and 3 h and 24 h after propacetamol administration (baseline, 3 h, and 24 h) at doses of 500 mg/kg, 1000 mg/kg, and 1500 mg/kg. We separated the serum fraction by centrifugation at 3000 rpm for 15 min. We isolated the serum component by centrifugation at 3000 rpm for 15 min. We quantified serum levels of aspartic aminotransferase (AST) and alanine aminotransferase (ALT) using a fully automated spectrophotometric method with an AU480 analyzer (Beckman Coulter, Inc., Brea, CA, USA) and commercial kits (Chema, Monsano, Italy).

### 4.7. Ex Vivo Real-Time PCR Analysis and Western Blotting Analysis

We sacrificed mice via CO_2_ inhalation 3 h after liver injury. We performed real-time PCR to measure the TSPO gene expression levels in the liver. We extracted the total RNA from liver tissue using the RNeasy Plus Mini Kit (Qiagen, Hilden, Germany) and converted 400 ng of RNA into complementary DNA using the Superscript^®^ III First-Strand Synthesis System (Invitrogen Corp., Carlsbad, CA, USA). We performed real-time PCR using the Powerup SYBR Green Master Mix (ABI; Thermo Fisher Scientific, Waltham, MA, USA) with 1 μL of complementary DNA and 0.2 μM primers on a QuantStudio 3 Real-Time PCR System (Applied Biosystems, Waltham, MA, USA) with the following parameters: 95 °C for 2 min, 40 cycles of 95 °C for 1 s, and 60 °C for 30 s. Relative expression levels were normalized to 18s ribosomal RNA expression. The primer sequences we used for the analysis are shown in Table S1 (see Supplementary Materials).

We performed Western blotting to measure the TSPO protein expression levels. We isolated total protein using a radioimmunoprecipitation assay buffer (Thermo Fisher Scientific). We separated the sample lysates by sodium dodecyl sulfate–polyacrylamide gel electrophoresis (SDS-PAGE) and transferred them to polyvinylidene difluoride (PVDF) membranes (Bio-Rad Laboratories, Hercules, CA, USA). The membranes were subsequently blocked with 5% non-fat dry milk (NFDM) for 1 h at room temperature and incubated overnight at 4 °C with primary antibodies targeting TSPO (#ab109497, diluted 1:1000; Abcam, Cambridge, UK) and β-actin (#ab8227, diluted 1:4000; Abcam). We then probed the membranes with horseradish peroxidase (HRP)-conjugated anti-rabbit (#7074; Cell Signaling Technology, Danvers, MA, USA) or anti-mouse IgG (#7076; Cell Signaling Technology). We measured signal intensities using a ChemiDoc MP system (Bio-Rad Laboratories).

### 4.8. Histological Analysis

We fixed liver tissue sections with 4% paraformaldehyde for one day and then washed them with water. We routinely processed the paraformaldehyde solution-fixed specimens and embedded them in paraffin wax. We cut 3 micrometer-thick sections and stained them with H&E for morphological evaluations.

For immunohistochemical staining, we blocked non-specific antibodies binding with 5% normal gout serum in Tris-buffered saline (TBS). We incubated sections with an anti-PBR antibody (#109497; Abcam) for the TSPO receptor at 4 °C overnight and then with a Polink-2 plus HRP Broad Spectrum Kit (ORIGENE cat: D41-125, Rockville, MD, USA). We performed a heat-mediated antigen retrieval using Tris/EDTA buffer pH 9.0. We counterstained sections with hematoxylin and visualized them under an inverted Olympus BX53 microscope (Olympus, Tokyo, Japan). We captured them with a QImaging camera and Image Pro 5.1 program. We quantified the TSPO-positive stained area in the IHC images using ImageJ software (version 1.54i, NIH, Bethesda, MD, USA). Briefly, the IHC images were first converted to 8-bit grayscale images. We then adjusted the threshold to highlight the TSPO-positive stained areas. We calculated the percentage of the total image area occupied by the TSPO-positive staining using the “Measure” function in ImageJ.

### 4.9. Statistics

All statistical analyses were performed using the MedCalc software package, version 12 (MedCalc Software, Ostend, Belgium) and GraphPad Prism 5.01 software (GraphPad Software, La Jolla, CA, USA). We used Mann–Whitney U tests to analyze the differences between two groups. For repeated measures (0, 3, and 24 h of hepatic injury), we used Friedman testing, which are non-parametric statistical tests for repeated measures. We used Kruskal–Wallis testing to analyze the differences among three groups. Statistical significance was set at *p* < 0.05. Data are expressed as median values with interquartile ranges.

## Data Availability

The data presented in this study are available from the corresponding author upon reasonable request.

## Supplementary Materials

The following supporting information can be downloaded at: https://www.mdpi.com/article/10.3390/ijms25115942/s1.
