# Supplementary material for: Application of TSPO-Specific Positron Emission Tomography Radiotracer as an Early Indicator of Acute Liver Failure Induced by Propacetamol, a Prodrug of Paracetamol
Source: Int J Mol Sci. 2024 May 29;25(11):5942. doi: 10.3390/ijms25115942 (PMC11173123; doi:10.3390/ijms25115942)
Supplement: Supplementary file 1 [file ijms-25-05942-s001.zip › ijms-3038808-supplementary.pdf]

Supplementary Materials

Application of TSPO-Specific Positron Emission Tomography Radiotracer as an Early Indicator of Acute Liver Failure Induced by Propacetamol, a Prodrug of Paracetamol

Daehee Kim [1,2], Hye Won Lee [1], Sun Mi Park [3], Ji Eun Lee [4], Sang Ju Lee [5], Bom Sahn Kim [3], Seung Jun Oh [5], Byung Seok Moon [3,*] and Hai-Jeon Yoon [4,*]

[1] Department of Emergency Medicine, Incheon St. Mary's Hospital, The Catholic University of Korea, Seoul 06591, Republic of Korea; kim_dae_hee@catholic.ac.kr (D.K.); joawony@naver.com (H.W.L.)
[2] Department of Emergency Medicine, College of Medicine, The Catholic University of Korea, Seoul 06591, Republic of Korea
[3] Department of Nuclear Medicine, Ewha Womans University Seoul Hospital, Ewha Womans University College of Medicine, Seoul 07804, Republic of Korea; psm9728@ewha.ac.kr (S.M.P.); kbomsahn@ewha.ac.kr (B.S.K.)
[4] Department of Nuclear Medicine, Ewha Womans University Mokdong Hospital, Ewha Womans University College of Medicine, Seoul 07985, Republic of Korea; qkraltnr60n@ewha.ac.kr
[5] Department of Nuclear Medicine, Asan Medical Center, University of Ulsan College of Medicine, Seoul 05505, Republic of Korea; atlas425@amc.seoul.kr (S.J.L.); sjoh@amc.seoul.kr (S.J.O.)
* Correspondence: bsmoon@ewha.ac.kr (B.S.M.); haijeon.yoon@ewha.ac.kr (H.-J.Y.)

Figure S1. Comparison the liver SUV between the control and acute liver failure group.

Figure S2. The full blot results of TSPO in Western blotting analysis.

Figure S3. The full blot results of β-actin in Western blotting analysis.

Table S1. Primer sequences used for real-time, reverse-transcriptase PCR.

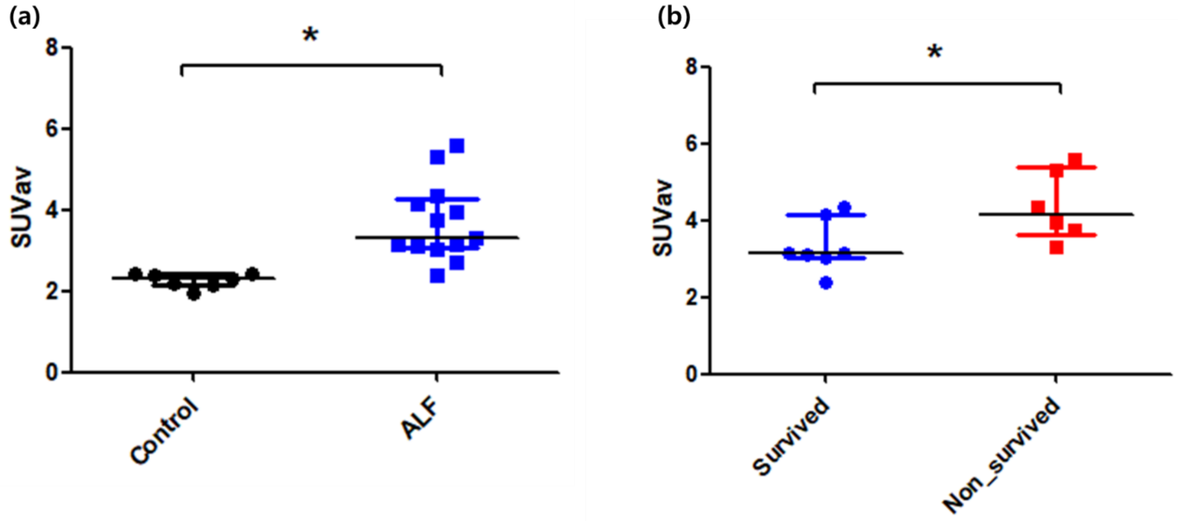

Figure S1. Comparison the liver SUV between the control and acute liver failure group. (a) A box plot comparing the liver SUV between the control and acute liver failure group. The median SUV is higher for the ALF group compared to the control group, indicating increased uptake in the liver of the ALF group; (b) A box plot comparing the liver SUV between survived and non-survived group. The median SUV is higher for the non-survived group compared to the survived group, suggesting that higher liver uptake is associated with a poorer outcome or non-survival; n=7 for control, n=7 for survived, and n=6 for non-survived; Data are expressed as median values with interquartile ranges; *p <0.05, **p <0.01 by Mann-Whitney test.

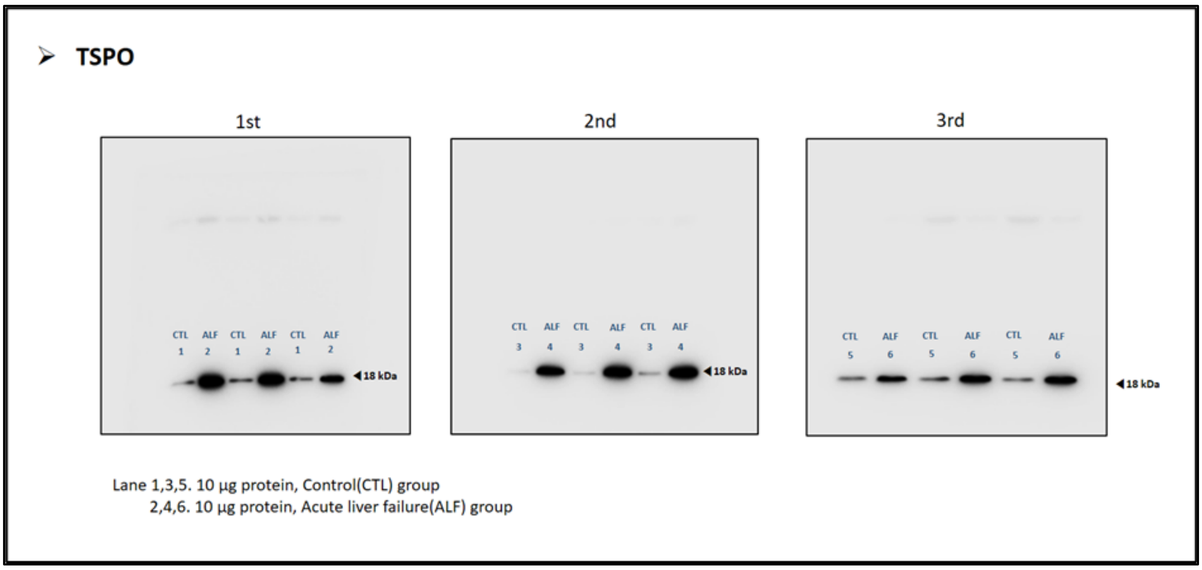

Figure S2. The full blot results of TSPO in Western blotting analysis (repeated three times in the control and acute liver failure group).

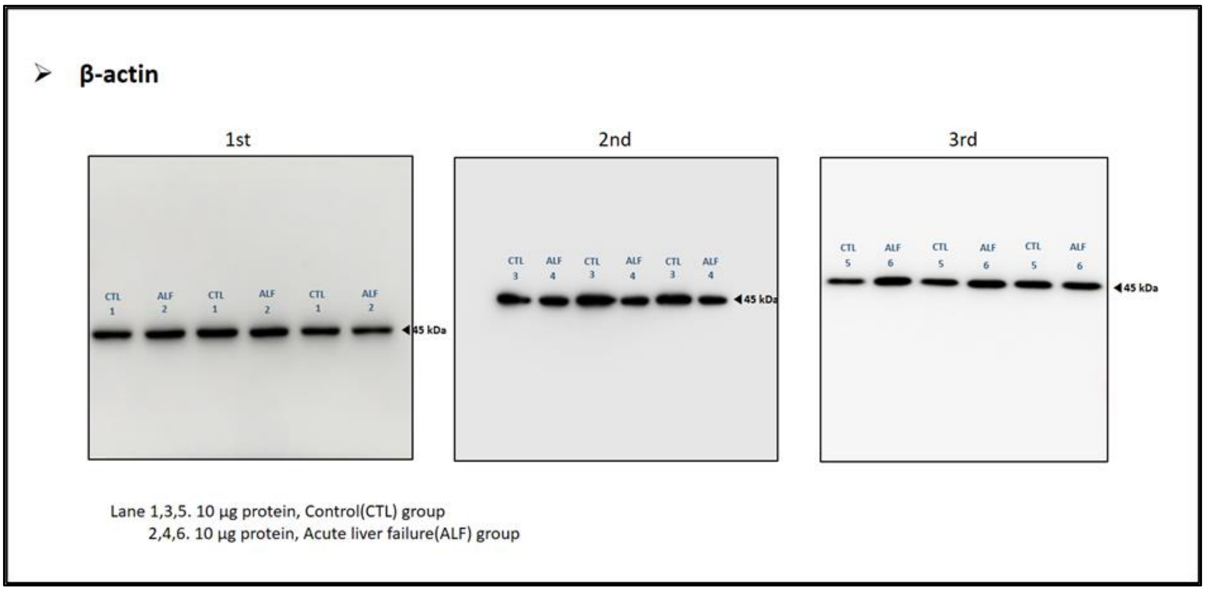

Figure S3. The full blot results of β-actin in Western blotting analysis (repeated three times in the control and acute liver failure group).

Table S1. Primer sequences used for real-time, reverse-transcriptase PCR.

Genes	Forward	Reverse	Size (bp)
TSPO	AGAAACCCTCTTGGCATCCG	CGTCCTCTGTGAAACCTCCC	120
18s rRNA	CGCTACACTGAACTGGCTCA	TGTGTACAAAGGGCAGGGAC	174
